# Interhemispheric symmetry of µ-rhythm phase-dependency of corticospinal excitability

**DOI:** 10.1038/s41598-020-64390-w

**Published:** 2020-05-12

**Authors:** Maria-Ioanna Stefanou, Dragana Galevska, Christoph Zrenner, Ulf Ziemann, Jaakko O. Nieminen

**Affiliations:** 1 Department of Neurology & Stroke, University of Tübingen, Tübingen, Germany; 2 Hertie Institute for Clinical Brain Research, University of Tübingen, Tübingen, Germany; 3 Department of Neuroscience and Biomedical Engineering, Aalto University School of Science, Espoo, Finland

**Keywords:** Motor cortex, Neuroscience, Motor control

## Abstract

Oscillatory activity in the µ-frequency band (8–13 Hz) determines excitability in sensorimotor cortex. In humans, the primary motor cortex (M1) in the two hemispheres shows significant anatomical, connectional, and electrophysiological differences associated with motor dominance. It is currently unclear whether the µ-oscillation phase effects on corticospinal excitability demonstrated previously for the motor-dominant M1 are also different between motor-dominant and motor-non-dominant M1 or, alternatively, are similar to reflect a ubiquitous physiological trait of the motor system at rest. Here, we applied single-pulse transcranial magnetic stimulation to the hand representations of the motor-dominant and the motor-non-dominant M1 of 51 healthy right-handed volunteers when electroencephalography indicated a certain µ-oscillation phase (positive peak, negative peak, or random). We determined resting motor threshold (RMT) as a marker of corticospinal excitability in the three µ-phase conditions. RMT differed significantly depending on the pre-stimulus phase of the µ-oscillation in both M1, with highest RMT in the positive-peak condition, and lowest RMT in the negative-peak condition. µ-phase-dependency of RMT correlated directly between the two M1, and interhemispheric differences in µ-phase-dependency were absent. In conclusion, µ-phase-dependency of corticospinal excitability appears to be a ubiquitous physiological trait of the motor system at rest, without hemispheric dominance.

## Introduction

The hypothesis that µ-oscillatory activity modulates cortical excitability in the sensorimotor cortex has recently received growing experimental support from electrophysiological studies in primates^1^ and humans^2–6^. The ongoing oscillations of motor networks at rest have been shown to resonate at the µ-frequency band (8–13 Hz)^7^, while the phase of the µ-rhythm has been related to a periodical transition between high- and low-excitability states in neurons of sensorimotor cortex^1^. In a study based on local field potential (LFP) recordings in the right monkey primary motor cortex (M1), neuronal firing rate was highest during the trough of the µ-oscillation, and lowest during its peak^1^.

The development of real-time electroencephalography (EEG)-triggered transcranial magnetic stimulation (TMS) has recently enabled a deterministic probing of the effects of the phase of a pre-stimulus oscillation on corticospinal excitability, as indexed by the motor evoked potential (MEP) amplitude, in the human M1. Using real-time brain-state-dependent TMS, we have shown that the EEG negative peak of the µ-oscillation represents a high-excitability state of corticospinal neurons (i.e., larger MEP amplitudes are elicited when TMS is triggered at the EEG negative peak of the µ-oscillation compared to the EEG positive peak)^2–5^. This effect has been demonstrated separately for the M1 of the motor-dominant^2,3^ and the motor-non-dominant^4^ hemisphere of right-handed subjects.

Although µ-oscillations are ubiquitously present in the sensorimotor cortex at rest^7,8^, significant interhemispheric differences in the frequency and power of µ-oscillations have been reported^9,10^. Electrophysiological studies (including LFP studies in primates^1^ and EEG–TMS studies in humans^2–5^) have assessed the effects of the pre-stimulus phase of µ-oscillations on M1 excitability unilaterally. We are unaware of electrophysiological evidence regarding the ubiquity (i.e., symmetry) of µ-phase-dependency of cortical excitability across the motor system (i.e., in homologous motor areas across hemispheres). Given the well-established, functional and structural interhemispheric asymmetries between homologous areas in the M1 cortices^11^, differences in the µ-phase-dependency of corticospinal excitability (e.g., in directionality or effect size) might be expected.

Hemispheric lateralization is a salient organizational feature of the motor system^12^. On the functional level, neuroimaging studies have indicated major differences in movement-associated activation patterns between the motor-dominant and motor-non-dominant hemispheres^13^, while TMS studies have shown significant interhemispheric differences between the cortical motor representation areas^14^ and the stimulation intensities required to elicit motor responses from the two M1 (with the motor-dominant hemisphere typically requiring lower stimulation intensities than the motor-non-dominant hemisphere)^15,16^. On the structural level, alongside interhemispheric differences in transcallosal^17^ and cortico-cortical M1 connectivity^18^, significant interhemispheric differences in local intracortical M1 circuits are noted^19^. For example, regional asymmetries of cortical thickness and local gyrification between the hand-knob areas of the two M1 have been associated with the degree of handedness, with evidence of robust leftward asymmetries (i.e., greater gyrification and cortical thickness in the motor-dominant M1) in consistent right-handers^20^. An increased intracortical connectivity within the motor-dominant M1 has also been supported by histological studies, which have shown increased neuropil concentrations (as an indirect measure of intracortical connectivity) within the motor-dominant compared to the motor-non-dominant M1^21^.

The motor output neurons in M1 are thought to be activated by TMS mainly transsynaptically through excitation of long-range cortico-cortical axons at the precentral and postcentral gyrus crowns, where the TMS-induced electrical field is strongest^22^. On the other hand, recent studies have shown that the cortical µ-oscillatory activity is regulated – alongside subcortical (i.e., thalamic) inputs – by local feedforward and feedback processes within superficial cortical layers in M1 (e.g., the supragranular pyramidal neurons^23,24^).

In the present study, we were interested in the possible asymmetries of µ-phase-dependency of corticospinal excitability, assessed via brain-state-dependent EEG-triggered TMS-determined resting motor threshold (RMT), between the homologous hand areas of the two M1 in consistently right-handed healthy subjects. To this end, we examined the interhemispheric correlation of the µ-rhythm phase effects (PHASE) on RMT between the two M1, assessing to what extent the µ-oscillation-dependency of corticospinal excitability may be regarded as a ubiquitous physiological trait of the motor system at rest or, alternatively, shows significant hemispheric asymmetry.

## Results

All procedures were well tolerated and no adverse events were noted. Based on the predefined inclusion criteria (see Methods), 51 (54%) out of 95 screened subjects were included in this study.

### RMT in different pre-stimulus µ-phase conditions

#### Motor-dominant primary motor cortex

**Figure 1:**
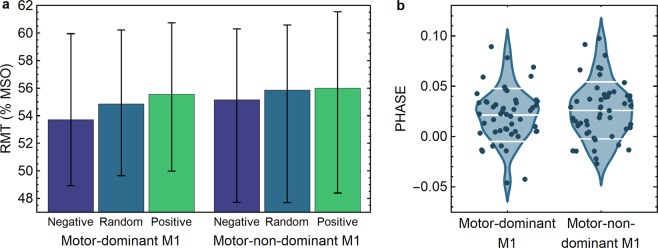
RMT and PHASE for the different stimulation conditions. (**a**) RMT as a percentage of the maximum stimulator output (%MSO). The bars represent the median over subjects and the whiskers indicate the interquartile ranges. (**b**) PHASE = (RMT_pos_ − RMT_neg_)/RMT_rand_ for the motor-dominant and motor-non-dominant hemisphere. The dots show the data of the individual subjects. The white bars indicate the mean and the mean ± standard deviation of the data.

RMT differed significantly depending on the pre-stimulus phase of the µ-rhythm, χ^2^(2)=34.756, *p* < 0.001 (Fig. 1). Pairwise comparisons were performed with a Bonferroni correction for multiple comparisons (statistical significance was accepted at *p* < 0.017). *Post hoc* analyses showed:

- A statistically significant difference between the negative-peak [median RMT_neg_, interquartile range (IQR): 53.70, (11.15)] and the random-phase condition [median RMT_rand_, (IQR): 54.85, (10.65)], *p* = 0.001.
- A statistically significant difference between the negative-peak and the positive-peak condition [median RMT_pos_, (IQR): 55.55, (11.05)], *p* < 0.001.
- A statistically significant difference between the random-phase and the positive-peak condition, *p* = 0.009.

#### Motor-non-dominant primary motor cortex

RMT differed significantly depending on the pre-stimulus phase of the µ-rhythm, χ^2^(2) = 34.402, *p* < 0.001 (Fig. 1). Pairwise comparisons were performed as above with accepted significance at *p* < 0.017. *Post hoc* analyses showed:

- A statistically significant difference between the negative-peak [median RMT_neg_, (IQR): 55.15, (12.70)] and the random-phase condition [median RMT_rand_, (IQR): 55.85, (12.95)], *p* = 0.003.
- A statistically significant difference between the negative-peak and the positive-peak condition [median RMT_pos_, (IQR): 56.00, (13.45)], *p* < 0.001.
- A statistically significant difference between the random-phase and the positive-peak condition, *p* = 0.005.

### No interhemispheric differences in RMT for each pre-stimulus µ-phase condition

Although the median RMT values of the motor-non-dominant hemisphere appeared larger than those of the motor-dominant hemisphere (Fig. 1a), Wilcoxon signed-rank tests did not show statistically significant interhemispheric differences for the positive-peak (*Z* = − 0.290, *p* = 0.772), negative-peak (*Z* = − 0.043, *p* = 0.965), or random-phase (*Z* = − 0.037, *p* = 0.970) conditions.

### No interhemispheric differences in PHASE

Two-tailed paired t-test did not show a significant difference between the PHASE in the motor-dominant M1 [mean, (SD): 0.022, (0.025)] and the motor-non-dominant M1 [0.025, (0.029)], *t*(50) =−1.124, *p* = 0.267; Cohen’s d = 0.11 (Fig. 1b). The corresponding Bayes factor was 0.276, indicating moderate evidence for PHASE being similar across the hemispheres.

### Significant positive correlation of PHASE in the motor-dominant M1 and PHASE in the motor-non-dominant M1

**Figure 2:**
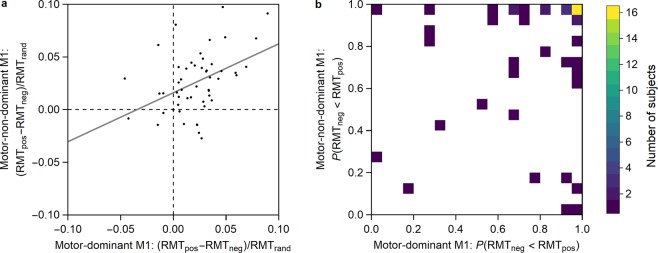
RMT differences for the motor-dominant and motor-non-dominant M1. (**a**) Difference in RMT for each subject between the µ-phase positive-peak and negative-peak conditions. The gray line shows a linear fit to the data. For 40/51 of the subjects (78%), either RMT_neg_ < RMT_pos_ (37/51 subjects, 73%) or RMT_pos_ < RMT_neg_ (3/51 subjects, 6%) was observed in both hemispheres, i.e., their data point is either in the upper right or lower left quadrant of the plot, respectively. **(b)** Number of subjects having a certain probability for RMT_neg_ < RMT_pos_ for the motor-dominant and motor-non-dominant M1. For each subject, the probabilities are obtained from their RMT probability density functions (see Methods, Fig. 3). The number of subjects is reported for adjacent 0.05 × 0.05 squares. The white region marks the area with no subjects.

A Pearson product-moment correlation indicated a positive correlation between PHASE in the motor-dominant M1 and PHASE in the motor-non-dominant M1, which was statistically significant (*r* = 0.432, *n* = 51, *p* = 0.002) (Fig. 2a).

**Figure 3:**
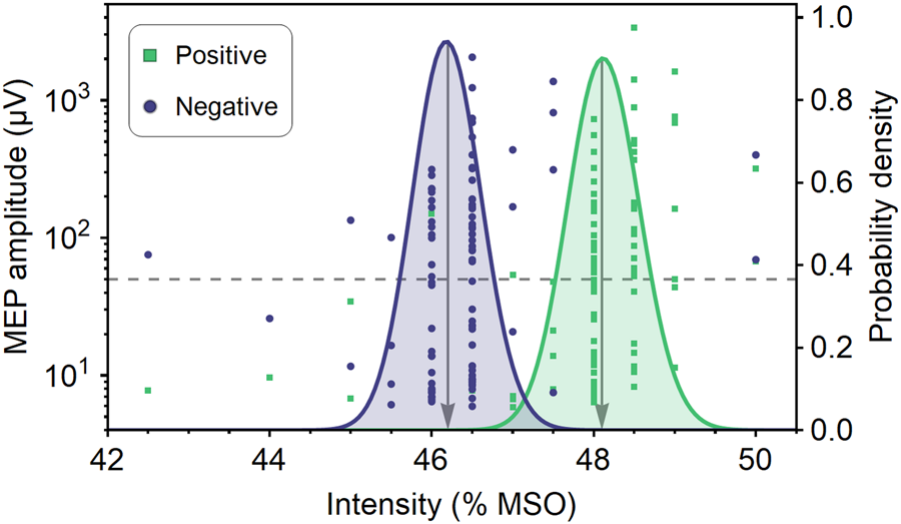
Resting motor threshold. MEP data of a representative single subject for µ-phase positive-peak and negative-peak conditions and the corresponding probability density functions for the RMT. The dashed line indicates the 50 µV MEP amplitude threshold. The arrows indicate the RMT that corresponds to the location of the maximum of the probability density function.

### Prevalence of PHASE effects in the motor-dominant vs. motor-non-dominant M1

Of the 51 right-handed subjects participating in this study RMT_neg_ < RMT_pos_ was observed

- In both the motor-dominant and motor-non-dominant M1 for 37 subjects (73%),
- In the motor-dominant M1 for 43 subjects (84%),
- In the motor-non-dominant M1 for 41 subjects (80%).

### Probability for RMTneg < RMTpos for the motor-dominant and motor-non-dominant M1

The observed differences in the RMT between the µ-phase positive-peak and negative-peak conditions (Fig. 2a) were associated with corresponding probabilities (Fig. 2b). Of the 51 subjects, for 16 subjects (31%) *P*(RMT_neg_ < RMT_pos_)>0.95 was observed for both the motor-dominant and motor-non-dominant M1, for 24 subjects (47%) *P*(RMT_neg_ < RMT_pos_)>0.95 for the motor-dominant M1, and for 27 subjects (53%) *P*(RMT_neg_ < RMT_pos_)>0.95 for the motor-non-dominant M1 (Fig. 2b).

### No correlations between interhemispheric differences in RMT, µ-rhythm peak frequency, or signal-to-noise ratio and the interhemispheric difference in PHASE

The rationale of these exploratory analyses was twofold: 1) As stronger brain-state-dependent modulation of corticospinal excitability occurs at weaker stimulation intensities^6^, interhemispheric differences in PHASE could be associated with interhemispheric RMT differences (i.e., differences in stimulation intensities). 2) The real-time phase targeting was based on a fixed frequency band of 8–13 Hz and C3/C4-centered Laplacian spatial filters for the extraction of the sensorimotor μ-rhythm. Thus, we were interested in assessing whether interhemispheric dissimilarities of PHASE could be associated with interhemispheric differences in µ-rhythm peak frequencies or µ-rhythm signal-to-noise ratio (SNR).

We did not find any significant correlations between the interhemispheric difference in PHASE and the interhemispheric differences in RMT (*r* = 0.019, *n* = 51, *p* = 0.896), µ-rhythm peak frequencies (*r* = −0.172, *n* = 51, *p* = 0.227), or µ-rhythm SNR (*r* = 0.191, *n* = 51, *p* = 0.176).

Out of the 51 subjects, 10 had dissimilar PHASE (i.e., *P*(RMT_pos_ > RMT_neg_)>0.5 for one hemisphere and <0.5 for the other), whereas 41 had similar PHASE (i.e., *P*(RMT_pos_ > RMT_neg_)>0.5 or <0.5 for both hemispheres). Subjects with dissimilar PHASE and subjects with similar PHASE across hemispheres had comparable interhemispheric differences of RMT [median, (IQR): 5.5, (6.5) vs. 4.4 (5.5), *U* = 194, *p* = 0.794], µ-rhythm peak frequencies [median, (IQR): 0.1, (0.3) vs. 0.2 (0.4), *U* = 160, *p* = 0.259], and µ-rhythm SNR [median, (IQR): 1.9, (1.8) vs. 1.6 (2.7), *U* = 195, *p* = 0.812].

### No interhemispheric differences in pre-stimulus signal power for each pre-stimulus µ-phase condition

Wilcoxon signed-rank tests did not show statistically significant interhemispheric differences of the pre-stimulus signal power in the µ-band for the positive-peak (*Z* = − 0.562, *p* = 0.574), negative-peak (*Z* = − 0.131, *p* = 0.896), or random-phase (*Z* = − 0.187, *p* = 0.851) conditions.

## Discussion

In this study, we investigated interhemispheric differences in µ-rhythm phase-dependency of corticospinal excitability between the hand-representation areas of the two M1 in 51 consistently right-handed human subjects, using real-time EEG-triggered TMS. We found no interhemispheric asymmetries in the directionality or effect size of µ-phase-dependency of corticospinal excitability. In particular, our results show a significant positive correlation between the µ-phase-dependency of corticospinal excitability of the motor-dominant and motor-non-dominant M1, and no differences in the extent of this effect between the two hemispheres. In accord with previous studies^2–6^, we corroborated that the negative peak of the µ-oscillation corresponds to a high-excitability state of the motor cortices, as reflected by the lower RMT values in the negative-peak condition compared to the positive-peak condition. These findings add to the previous results, as RMT was used here for the first time as a readout of corticospinal excitability, whilst in all previous studies the effect of pre-stimulus phase of µ-oscillations on corticospinal excitability had been assessed by large-amplitude MEPs^2–6^. This is an important methodological difference, as small MEPs around RMT are generated by different mechanisms than large MEPs, which are elicited by clearly suprathreshold stimulation intensities. Small MEPs originate predominantly from local monosynaptic activation (i.e., they are reflected in I1-waves), whereas large MEPs are produced by polysynaptic activation through long-range cortico-cortical and subcortical–cortical projections (i.e., they are reflected in late I-waves)^25,26^. Therefore, the observed changes in RMT reflect more closely the µ-phase effects on the excitability of the corticospinal neuron *per se*, as opposed to the previously observed changes in large MEP amplitudes, which may reflect alterations in an extended sensorimotor network.

In the present study, we also systematically investigated the prevalence of µ-phase-dependency of corticospinal excitability in the so-far largest cohort of consecutively included healthy right-handers, and found that 73% of the included subjects (based on the pre-defined exclusion criteria described in Methods) presented phase-dependency in the form of RMT_neg_ < RMT_pos_ in both hemispheres, while 84% and 80% of the subjects presented this phase-dependency in the motor-dominant and motor-non-dominant hemisphere, respectively. These high prevalences are compatible with our previous observation that the µ-phase effect is most strongly expressed in MEPs of small amplitude^2–6^.

With regard to the motor cortices, it is known that, between hemispheres, significant macroanatomical^20,27^ and microanatomical^21^ side differences exist, with a considerable degree of inter-individual variability^28^. Interhemispheric differences in neuronal circuitry have been developmentally related to functional lateralization^29^, and in particular to the direction and degree of handedness^30^. Morphologically, the pyramidal tract presents significant leftward asymmetries in right-handers, with larger volume of descending cortical motor fibers on the left side, at cortical^28^ and subcortical level^31^. Hence, it is an implicit assumption that interhemispheric differences in corticospinal excitability, as suggested by electrophysiological studies^15^, majorly rely on structural–functional differences in homologous motor pathways. In accord with previous electrophysiological studies^16^, our results indicated that RMT values of the motor-non-dominant hemisphere were on average larger than those of the motor-dominant hemisphere; however, these differences did not reach statistical significance, possibly due to methodological aspects, such as the use of a biphasic rather than monophasic current waveform^32^. Given these structural–functional leftward asymmetries in right-handers, we addressed whether stronger µ-phase-dependency of corticospinal excitability could occur when stimulating the motor-dominant compared to the motor-non-dominant M1. However, our results do not support this hypothesis, as no interhemispheric asymmetries in the µ-phase-dependency of corticospinal excitability were noted. These findings, in conjunction with the evidence of a significant positive correlation between the µ-phase-dependency of cortical excitability between the two primary motor cortices, support the proposition that µ-phase-dependency of cortical excitability is an inherent, ubiquitous trait of the motor system at rest.

We acknowledge possible limitations of the present study. First, the order of the tested hemispheres was not randomized, which might have rendered the conditions of two hemispheres dissimilar. Nonetheless, after single-pulse TMS and a delay between the testing of the two M1 in the range of several minutes, no sustained TMS-induced modulation of brain activity in the contralateral hemisphere would be expected^33^. Second, the real-time phase targeting was based on a fixed frequency band of 8–13 Hz and C3/C4-centered Laplacian spatial filters for the extraction of the sensorimotor μ-rhythm. Although the use of individualized frequency bands and spatial filters might have improved our results^5^, we showed that the dissimilarities of µ-phase effect (noted in 10 out of 51 subjects between the two M1) were not associated with interhemispheric differences in µ-rhythm peak frequencies or SNR. Third, due to the predefined inclusion criteria of this study (i.e., including only right-handers with prominent sensorimotor µ-rhythm, and excluding subjects with a high motor threshold), further research is warranted to assess the generalizability of the present results.

In conclusion, we found no interhemispheric asymmetries in the directionality or effect size of µ-phase-dependency of corticospinal excitability between the two primary motor cortices. A high prevalence of µ-phase-dependency of cortical excitability was noted, with 73% of the 51 tested subjects having higher RMT in the pre-stimulus µ-positive-peak condition as opposed to the µ-negative-peak condition in both M1. Our findings suggest that µ-phase-dependency of cortical excitability is an inherent, symmetrical trait of the motor system at rest.

## Methods

### Participants

The study was approved by the local ethics committee of the medical faculty of the University of Tübingen (protocol 716/2014BO2). The experiments were performed in accordance with the Declaration of Helsinki and current TMS safety guidelines^34^. Written informed consent was obtained from all participants prior to their participation. Ninety-five healthy volunteers without a history of neurological or psychiatric disease or use of central nervous system active drugs, alcohol or nicotine were screened to identify 51 (54% of the screened) subjects (29 female and 22 male; mean age ±1 SD: 24 ± 6 years, age range: 18–61 years; all right-handed according to the Edinburgh handedness inventory^35^, mean ±1 SD laterality score: 74 ± 26; range: 27–100) that fulfilled the following inclusion criteria: (i) TMS-evoked movement of the left first dorsal interosseous (FDI) muscle at ≤80% of the maximum stimulator output (MSO) intensity; (ii) SNR of the μ-band (8–13 Hz) EEG signal ≥5 dB on the left and right sensorimotor cortices, respectively (with the subject at rest and eyes open; see below for more details)^36^. This criterion ensured that the µ-rhythm was strong enough to enable our algorithm to estimate the instantaneous phase of the EEG signal with sufficient accuracy^2,37^. Twenty-six of the invited 95 subjects failed on the FDI-movement criterion, and 18 on the SNR criterion.

### Experimental set-up

We recorded EEG with a TMS-compatible Ag/AgCl sintered ring electrode cap (EasyCap GmbH, Germany) with electrodes at the locations C3, C4, FC1, FC2, FC5, FC6, CP1, CP2, CP5, and CP6 of the 10–20 International system^38^. The reference and ground electrodes were at the locations FCz and POz, respectively. The EEG data were sampled with a 24-bit biosignal amplifier (low-pass filtering at 1250 Hz, DC mode, 5-kHz sampling rate; NeurOne Tesla, Bittium Biosignals Ltd., Finland). EMG was recorded from the left and right FDI with adhesive hydrogel electrodes (Kendall, Covidien, Ireland) in a belly–tendon montage connected to the amplifier recording also the EEG (0.16–1250-Hz bandpass filter, 5-kHz sampling rate).

TMS was administered with a figure-of-eight coil (70-mm winding diameter; PMD70-pCool, MAG & More GmbH, Germany) driven by a PowerMAG stimulator (PowerMAG Research 100 ppTMS, MAG & More GmbH). The applied pulse waveforms were biphasic (160-µs period) with the second, biologically predominantly effective phase of the induced electric field in the cortex being in the lateral–posterior to medial–anterior direction. The stimulation intensity was set through a serial-port connection^39^. The coil position was monitored throughout the experimental session with a neuronavigation system (Localite TMS Navigator, Localite GmbH, Germany) and adjusted if necessary. The subject’s head was registered to a standard Montreal Neurological Institute head magnetic resonance image. A vacuum pillow (Vacuform 2.0 surgical cushion, B.u.W. Schmidt GmbH, Germany) and a fixation arm (Super Flex Arm (long), Tonica Elektronik A/S, Denmark) were used to immobilize the head and to maintain a fixed coil placement.

EEG data were preprocessed in real time with a Simulink Real-Time model (R2016a, The MathWorks, Inc., MA, USA) running on a dedicated xPC Target computer with the Simulink Real-Time operating system^2^. The model analyzed the ongoing EEG signals provided by the analog output terminal of the amplifier (amplified, 5-kHz sampling rate, 1250-Hz low-pass filter). The EEG activity corresponding to the left (motor-dominant) and right (motor-non-dominant) M1 was extracted from a Laplacian montage centered at channel C3 (channel C3 minus the mean of the surrounding channels FC1, FC5, CP1, and CP5) and at channel C4 (channel C4 minus the mean of the surrounding channels FC2, FC6, CP2, and CP6), respectively^37^. Another parallel Simulink Real-Time model running on the xPC Target computer controlled the triggering of the TMS device based on the EEG data of the Laplacian montages. First, 500-ms-long sliding windows of the data were forward and backward filtered with a finite impulse response filter (8–13-Hz bandpass). Then, the coefficients for an autoregressive Yule–Walker model (order 30) using 372 ms of the filtered data in the middle of the sliding window were calculated. With the autoregressive model, a 128-ms-long prediction of the signal, centered at the end of the original sliding window, was generated. From this prediction, the instantaneous phase of the signal was estimated by computing an analytic signal with the Hilbert transformation. The TMS device was triggered when a targeted µ-rhythm phase was met, provided that the signal power in the 8–13-Hz band exceed a predetermined threshold. Further details of the real-time system are provided in previous publications^2–6^.

### Experimental sessions

The experiment started with a 10-min recording of resting-state EEG (eyes open, subject instructed to relax and fixate to a cross at eye level in front of them). If the analysis of the resting-state-EEG data indicated a sufficient signal power in the µ-frequency band in both hemispheres, we proceeded with single-pulse TMS. During TMS, subjects were instructed to keep their hands relaxed and their eyes open and to fixate to a cross in front of them. First, we identified the right-FDI hotspot in the left (motor-dominant) M1 as the coil position and orientation resulting, at a slightly suprathreshold stimulation intensity, in maximal MEP amplitudes^25^. Then, we placed the coil at the hotspot and, with a threshold-tracking technique^40^, determined RMT, i.e., the minimum stimulation intensity that would evoke MEPs exceeding 50 µV in peak-to-peak amplitude in 50% of the trials for the right FDI in three conditions: positive peak, negative peak, or random phase of the µ-oscillation in the C3 Laplacian. The threshold tracking was conducted independently for each condition. A Matlab (The MathWorks, Inc.) program detected the peak-to-peak MEP amplitude and adjusted the next stimulation intensity for the corresponding condition according to the threshold-tracking algorithm to match the current RMT estimate. In each of the three µ-phase conditions, we administered 100 stimuli at the intensities suggested by the threshold-tracking algorithm. In accord with previous work^3,6^, the administration of 100 stimuli per µ-phase condition was chosen to ensure an adequate number of trials (i.e., after trial removal during the EEG and EMG preprocessing stages) to achieve sufficient statistical power for differentiating phase-specific stimulation effects. The stimulation order was pseudorandomized so that blocks of three consecutive stimuli contained one stimulus targeting each µ-phase condition. The minimum interstimulus interval was 2 s. Finally, we conducted an otherwise identical single-pulse TMS experiment on the right M1 with EMG recording from the left FDI and with stimulus conditions based on the real-time-analyzed EEG signal from the C4 Laplacian.

In contrast to our previous studies^2–6^, we tested here RMT rather than MEP amplitude as a marker of corticospinal excitability. The rationale is twofold: (1) We have shown previously that the µ-phase effect on corticospinal excitability is predominantly expressed in small-amplitude MEPs, i.e., close to motor threshold;^2–6^ (2) Small MEPs are generated by different mechanisms than large MEPs. The small MEPs originate predominantly from local monosynaptic activation (i.e., they are reflected in I1-waves), whereas large MEPs are produced by polysynaptic activation through long-range cortico-cortical and subcortical–cortical projections (i.e., they are reflected in late I-waves)^25,26^. Therefore, changes in RMT will reflect more closely the µ-phase effects on excitability of the corticospinal neuron *per se*, compared to changes of large MEP amplitudes that would reflect alterations in an extended sensorimotor network.

### Data preprocessing

EEG and EMG data were analyzed offline with custom-made Matlab (R2016a or newer) scripts. We calculated the power spectrum of the resting-state EEG data from the C3- and C4-Laplacian signals, respectively, and estimated the SNR of the µ-frequency band signal as the peak of the power spectrum in the 8–13-Hz band after subtracting the 1/f component^5^. The peak frequency was saved for further analysis. The EEG data of the left M1 and right M1 TMS sessions of each subject were analyzed as follows: (1) The C3/C4-Laplacian signals were epoched with respect to the TMS pulses and the average of the signal in a 600-ms-long baseline window immediately preceding the TMS pulse was subtracted from them. (2) The baseline signals of each subject were plotted on top of each other and an individual threshold was visually identified (due to the variable signal characteristics across subjects, the threshold was based on the judgement of the person conducting the analysis) for each subject (separately for the C3/C4-Laplacian signals). (3) Trials with a baseline exceeding the individual threshold were discarded from the subsequent analysis to exclude trials in which noise/artifacts might have compromised the phase estimation.

The EMG data were high-pass filtered at 5 Hz by applying a second-order Butterworth filter in the forward and backward direction, respectively. The EMG data were then epoched with respect to the TMS pulses and baseline-corrected, with the baseline window ranging from −200 to 0 ms with respect to the TMS pulse. The TMS artifact was removed by linearly interpolating the signal between 0 and 10 ms, and the signal was filtered with a second-order Butterworth notch filter (49–51 Hz; applied in both directions) to reduce power-line noise. A fifth-order polynomial was fitted to and subtracted from the baseline data to reduce the effect of a TMS-pulse-related signal drift on the baseline data. The data were then baseline corrected for the second time. Trials containing muscle activation, artifacts, or noise exceeding an individual threshold in a 200-ms period preceding the TMS pulse were discarded, since pre-innervation increases the MEP amplitude^41^. These individual EMG thresholds for the left and right FDI, respectively, were determined visually by plotting the baseline EMG traces on top of each other (due to the variable signal characteristics across subjects, the thresholds were based on the judgement of the person conducting the analysis). In total, 17% of the trials were discarded. Only 1% of the trials were discarded based on the EEG criteria, and the remaining trials were discarded due to insufficient EMG quality.

### Data analysis

The data passing the EEG and EMG preprocessing stages were used for subsequent analysis. For each accepted trial, we determined the MEP peak-to-peak amplitude in a time window of 15–45 ms with respect to the TMS pulse. For each phase condition and subject, we fitted cumulative Gaussians to the MEP data corresponding to the left M1 and right M1 stimulation, respectively^40^. These cumulative Gaussians modeled the probability of obtaining an MEP exceeding 50 µV in peak-to-peak amplitude and allowed us to obtain the probability density for RMT as a function of stimulation intensity (Fig. 3)^40^. The RMT of the right and left FDI, serving as a measure of the corticospinal excitability of the left M1 and right M1, respectively, was identified as the location of the maximum of the corresponding probability density function. We also determined for each subject and hemisphere the probability that the RMT in the positive-peak phase condition was higher than the RMT in the negative-peak phase condition (i.e., *P*(RMT_pos_ > RMT_neg_)) based on the probability density functions. This was achieved by integrating the joint probability density function (defined for stimulation intensities 0–100% MSO) over the triangular area in which the stimulation intensity for the positive-peak phase condition exceeded the intensity for the negative-peak phase condition.

To assess whether the pre-stimulus signal power in the µ-band was similar across hemispheres in all three conditions of phase-dependent RMT estimation, we calculated the average pre-stimulus power for each subject adhering to a previously published pipeline^42^. The following steps were performed: 1) For each accepted trial, we extracted 2 seconds of the C3/C4-Laplacian EEG data centered around the time of the TMS pulse (time 0 ms). 2) We removed the TMS artefact by linearly interpolating the signal from 0 to 15 ms. 3) After applying a second-order Butterworth anti-aliasing filter (cut-off frequency 200 Hz) in forward and backward directions, we downsampled the data to 500 Hz. 4) We estimated the power spectral density with the Burg’s method (model order 26; 1-Hz frequency resolution; frequency range: 1–45 Hz) in the time window ranging from −150 to 0 ms with respect to the TMS pulse. 5) After subtracting the 1/f component^5^, we extracted the total µ-band power at 8–13 Hz.

### Statistical analysis

Statistical analyses were performed with IBM SPSS Statistics v.23 (IBM, NY, USA) and Matlab (The MathWorks, Inc.). The distribution of data was checked with the Shapiro–Wilk test; normally and non-normally distributed data were analyzed with parametric and non-parametric tests, respectively. Differences in the RMT for the different pre-stimulus µ-phase conditions, i.e., the negative (neg) and positive (pos) peaks of a Laplacian signal and the random phase (rand), were assessed using Friedman tests (due to non-normal distributions). Wilcoxon signed-rank tests were used to assess the interhemispheric differences in RMT for each condition. Also, Wilcoxon signed-rank tests were used to assess possible interhemispheric differences in the average pre-stimulus signal power for each condition. We defined PHASE as (RMT_pos_ − RMT_neg_)/RMT_rand_. To assess whether PHASE in the left M1 was correlated with PHASE in the right M1, Pearson’s correlation analysis was performed (after confirming linear relationship between the variables, and normal distribution of the data). A two-tailed paired t-test was used to assess differences in PHASE between hemispheres (in addition to the *p* value, we calculated also the corresponding Bayes factor with the bayesFactor Matlab toolbox^43^). The prevalence of PHASE and probabilities for RMT_neg_ < RMT_pos_ in each hemisphere were also calculated. To assess whether interhemispheric differences of PHASE were correlated with interhemispheric differences of RMT (i.e., RMT_rand_), µ-rhythm peak frequencies, or SNR, Pearson’s correlation analyses were performed. In an exploratory analysis, interhemispheric differences in RMT, µ-rhythm peak frequencies, and SNR were assessed in subjects with *P*(RMT_pos_ > RMT_neg_) > 0.5 for one hemisphere and <0.5 for the other (i.e., dissimilar PHASE across hemispheres) vs. subjects with *P*(RMT_pos_ > RMT_neg_) > 0.5 or <0.5 for both hemispheres (i.e., similar PHASE across hemispheres) using Mann-Whitney U tests (due to non-normal distributions). The significance level was set for all statistical tests to <0.05 (unless stated otherwise).

## Data Availability

Data are available from the corresponding author upon reasonable request.
